# Association of thermal perceptions, metabolic rate, clothing, and local skin temperature in people with cold constitution in air-conditioned office environments

**DOI:** 10.1186/s40101-025-00407-5

**Published:** 2025-11-06

**Authors:** Biplob Kanti Biswas, Koichi Ishii, Yu Watanabe, Jiating Li, Yumiko Tan, Ayano Dempoya, So Takeuchi, Sang-il Lee, Takuji Iwamura, Shingo Konoshita, Hitoshi Wakabayashi

**Affiliations:** 1 Laboratory of Environmental Ergonomics, Faculty of Engineering, Hokkaido University, N13 W8, Kita-ku, Sapporo, Hokkaido 060-8628 Japan; 2 Taisei Corporation, Shinjuku-ku, Tokyo Japan

## Abstract

Cold constitution refers to a phenomenon in which individuals have a higher sensitivity to cold and feel colder than others. This research aimed to examine the associations of morphological characteristics, personal factors, thermal perceptions, and local skin temperature (*t*_*sk*_) with cold constitution by conducting a field experiment. It also explored differences in these aspects between individuals with and without cold constitution, in a thermoneutral office environment during summer and winter, and in 89 and 75 sedentary workers, respectively. A questionnaire survey was conducted to classify the cold constitution (CC) and non-cold constitution (NC) groups. The results indicated that females and individuals with lower body mass index (*BMI*) were more likely to have cold constitution. The CC group exhibited a significantly lower metabolic rate (*M*) in both seasons, lower thermal sensation votes, warmer thermal preference, and a greater predicted percentage of dissatisfied in summer (*p* < 0.01). No significant differences were observed in clothing insulation between the groups; however, winter clothing was significantly higher compared to summer for both groups (*p* < 0.01). Furthermore, the CC group exhibited significantly lower local skin temperatures at distal body parts (*p* < 0.01). Significant correlations were observed for gender, *BMI*, *M*, thermal sensations, and distal *t*_*sk*_ with cold constitution. Adjusting the effects of gender and *BMI*, most correlations with cold constitution weakened. However, thermal sensation remained significant in summer, while no correlation was observed with *t*_*sk*_. These findings emphasize the significant associations of morphological characteristics, personal factors, and thermal perceptions with cold constitution and show the importance of assessing the thermal environment.

## Introduction

In modern society, the majority of individuals spend around 8 to 9 h a day in office environments, as part of their routine work. The thermal condition of these office environments has enormous impacts on their mental health, physical comfort, and work efficiency [1, 2]. The thermal environment within those conditions plays a vital role in shaping individuals’ experiences and productivity [3]. Occupants’ performance, proficiency, and concentration are also associated with the thermal environmental conditions [4]. Human cognitive performance, such as memory recall tasks, attention like vigilance, reaction time, visual and motor tracking, and motor coordination, can be altered significantly in different climatic conditions [5–7]. This change in performance is driven by the alteration of psychological and/or biological responses [4].

Furthermore, the design and management of the environment inside the office spaces can either enhance or hamper employees’ well-being [8]. Previous studies have also reported that the office environment has a significant impact on office workers’ performance and their comfort, and a comfortable environment can enhance their performance [9, 10]. To address these issues, most of the offices try to keep their air-conditioning system in a neutral condition where most of the users can experience thermal comfort (TC) and neutral thermal sensation.

Thermal comfort and thermal sensation can be expressed as the human responses to thermal stimuli, which evoke specific sensory perceptions [11]. Thermal comfort is a subjective assessment of whether the thermal environment is satisfactory or acceptable, and it is influenced by physical, physiological, psychological, and many other processes [12]. The human mind makes the conclusion about comfort based on direct temperature and moisture sensations from the skin, the temperature of the core body, and the necessary effort to balance body temperature [13, 14]. Comfort is conditional not only on environmental factors but also on how these conditions are perceived by human beings [15]. On the other hand, thermal sensation is influenced primarily by the activation of thermoreceptors on the skin surface and other sensory pathways that detect changes in the surrounding environment [16]. Thermal sensation refers to the immediate and conscious perception of a thermal environment, such as feeling hot or cold at various levels. Furthermore, thermal preference (TP) and thermal dissatisfaction level (DL) are additional parameters used to evaluate the thermal environment [17]. All these evaluations require individual surveys through personal inquiries. Interestingly, individuals’ morphological characteristics, including age, gender, body size, and clothing and activity level, influence their individual experience of thermal comfort, thermal sensation, thermal preference, and satisfaction differently under a given condition [18].

Furthermore, in a uniform and steady thermal environment, the predicted mean vote (*PMV*) is a widely used mathematical index that predicts human thermal sensation on the basis of environmental factors such as air temperature (*t*_*a*_), relative humidity (*RH*), mean radiant temperature (*t*_*r*_), and air velocity (*ν*_*a*_) with personal factors, specifically metabolic heat production (*M*) and clothing insulation (*I*_*cl*_) [19, 20]. It expresses thermal sensation via a 7-point assessment scale, ranging from cold (−3) to neutral (0) to hot (+3). According to ASHRAE, people feel comfortable within a *PMV* range of −0.5 to +0.5 [12]. Additionally, in this range, more than 90% of individuals are satisfied with the environmental conditions, which is acceptable for setting the thermal environment [20]. Furthermore, *PMV* prediction requires the consideration of personal factors. However, because of the challenging nature of personal data collection, in most cases, personal data is considered as common uniform values, leading to incorrect *PMV* predictions [21, 22]. Based on the *PMV* prediction, the predicted percentage of dissatisfied (*PPD*) is another parameter used to estimate the percentage of subjects likely to be dissatisfied with a given environmental setting. *PPD* prediction also depends on the thermal environment [23]. It was found that even vertical temperature differences in the same space can elevate dissatisfaction percentages [24]. For accurate estimation of *PPD*, it is essential to estimate the environmental factors and personal factors accurately.

Another important aspect of assessing human thermal sensation is the skin temperature (*t*_*sk*_). It is a direct physiological response of the body to the surrounding thermal environment [25]. The skin acts as the boundary layer between the body and the environment and plays a key role in the thermoregulation process as well as the heat balance [26]. Hot and cold receptors located just below the skin send signals through the sensory nervous system to the anterior hypothalamus, helping to regulate body temperature [27, 28]. This finding indicates that skin temperature reflects the impact of the thermal environment on individuals and has an intimate connection with thermal sensation and comfort.

Considering thermal comfort and sensation, even in the same thermal environment, different individuals can perceive the environment differently. Beyond environmental influences, individual characteristics also play a vital role in the perception of the thermal environment. Consequently, a common thermal setup may not be comfortable for everyone. This can be explained by individual differences, where individual characteristics as well as morphology have a considerable impact on thermal perception [29]. In previous studies on individual differences in thermal sensation and comfort, research focused primarily on factors like age, gender, and certain morphological characteristics such as *BMI* [30–32].

For example, in age-related studies compared with younger males, elderly males demonstrated reduced sensitivity in perceiving cutaneous warmth [33, 34]. In gender-related studies, females generally preferred slightly warmer environments for thermal comfort, with preferred temperatures approximately 1.0 to 2.5 °C higher than those preferred by males [35–37]. In *BMI-related* studies, it was observed that individuals with lower *BMI* were comfortable at a little bit higher ambient temperature [38], and lower *BMI* individuals are more prone to feeling cold [39].

However, there has been little research on individual differences associated with cold constitution, which is a significant individual difference that needs to be considered.

The term “cold constitution,” known as “hie-sho” in Japanese [40], originates from traditional East Asian medicine and culture. This refers to a condition where an individual is substantially sensitive to cold [41]. Individuals with cold constitution have a distinct skin blood flow regulatory system with heightened adrenergic sensitivity, leading to increased cutaneous vasoconstriction in the distal extremities during cold exposure [42]. Certain studies focusing on cold constitution have reported that people with cold constitution experience greater cold sensations even in the thermoneutral environment recommended by ASHRAE [43, 44]. This concept is related to the broader idea of maintaining balance in body temperature and overall health. Cold-sensitive people mostly feel cold sensations, particularly in extremities like hands and feet. This symptom was observed when they were exposed to a cold or even neutral environment, and they presented greater sympathetic nerve activity [44]. Inadequate blood flow to the extremities due to vasoconstriction can lead to persistent cold sensations [45]. Considering that to ensure thermal satisfaction for a long period of time, it is essential to consider the cold constitution while designing personal air-conditioning systems rather than just relying on a uniform air-conditioning environment for all.

Considering the facts, this research aimed to investigate the associations of morphological characteristics, gender, thermal perceptions, *M*, *I*_*cl*_, *PMV*, *PPD*, and local skin temperature with cold constitution in an air-conditioned office environment.

## Methods

This research was conducted in an office environment as a field experiment. The experiment was carried out in summer (2023 August 30–2023 September 05) and winter (2024 February 08–2024 February 14) among regular office employees. The research procedure was reviewed and approved by the IRB of the Faculty of Engineering, Hokkaido University (no. R5-8).

### Participants

Table 1 summarizes the morphological characteristics of the participants. In the summer, 89 participated in this experiment, while in the winter, there were 75 volunteers. All participants were briefed about the research protocols and provided written informed consent before their participation. They took part in the experiment during their regular office hours. During their participation, they were free to wear any type of clothing of their choice.

**Table 1:** Morphological characteristics and core body temperature of the participants

Morphology	Summer		Winter	
	CC group	NC group	CC group	NC group
Number of participants	29	60	24	51
Gender (M:F)	18:11	54:6	13:11	48:3
Age (year)	35.97 ± 11.57	36.32 ± 12.12	36.21 ± 11.07	37.51 ± 11.89
Height (cm)	166.98 ± 7.45	170.48 ± 6.57*	165.46 ± 7.17	170.45 ± 6.36*
Weight (kg)	59.62 ± 10.31	69.05 ± 10.99*	57.63 ± 11.09	68.37 ± 8.42*
*BMI* (kg/m^2^)	21.27 ± 2.62	23.67 ± 2.90*	20.91 ± 2.96	23.51 ± 2.45*
*t*_cr_ (°C)	36.64 ± 0.31	36.66 ± 0.30	36.70 ± 0.25	36.66 ± 0.29

Values are mean ± standard deviation, *p < 0.05 between CC group and NC group

*CC* cold constitution, *NC* non-cold constitution, *M* male, *F* female, *BMI* body mass index, *t*_cr_ armpit temperature as a substitute for core body temperature

### Research procedure

The experiment was conducted in a fully air-conditioned office in Tokyo, Japan, where the indoor thermal conditions were in the thermoneutral range recommended by ISO 7730 [20] (Table 2). The outdoor monthly mean air temperatures were 29.2 °C and 26.7 °C in August and September 2023 (summer), respectively, and 8 °C in February 2024 (winter) [46]. The outdoor monthly mean relative humidities were 78% and 80% in August and September 2023 (summer), respectively, and 63% in February 2024 (winter) [46]. The experiment took place on the 17th floor of a 54-story office building. The average floor area was approximately 3400 m^2^, with no partition except for the service zones. The building featured an RCC structure with double-glass window openings, and the windows had blinders designed to minimize solar heat gain. The centrally controlled air-conditioning system and open floor design ensured a stable indoor environment, efficiently minimizing the impact of outdoor conditions. To minimize the influence of external environmental conditions, the experiment was conducted after participants had spent at least 1 h engaged in regular office work, allowing for adaptation to the office thermal environment.

**Table 2:** Environmental conditions during the research period

Environmental condition	Summer	Winter
*t*_a_ (°C)	25.09 ± 0.28	25.52 ± 0.50
*t*_r_ (°C)	25.62 ± 0.26	25.72 ± 0.61
*RH* (%)	59.06 ± 1.71	36.10 ± 1.82
*ν*_a_ (m/s)	0.14 ± 0.00	0.11 ± 0.04
*p*_a_ (*P*_a_)	1924.7 ± 38.4	1477.7 ± 65.5
*h*_c_ (W/(m^2^∙K))	4.46 ± 0.07	4.06 ± 0.51
*h*_r_ (W/(m^2^∙K))	5.95 ± 0.02	5.95 ± 0.03

*t*_a_ air temperature, *t*_r_ mean radiant temperature, *RH* relative humidity, *ν*_a_ air velocity, *p*_a_ water vapor pressure in ambient air, *h*_c_ convective heat transfer coefficient, *h*_r_ radiative heat transfer coefficient

This research started with a questionnaire survey regarding cold constitution (Table 3). Then, the subjects’ morphological data, such as age, sex, height, and body weight, were collected through a questionnaire survey. After that, there was a 60-min adaptation to the thermal environment. During this period, the subjects were asked to perform their regular office work. There were no instructions to restrict their work patterns. Although their activities were mostly sedentary work, such as sitting while working on computers like typing, reading and writing documents, and organizing materials like documents. At the end of the experiment, thermal voting was performed through a subjective questionnaire. The armpit temperature was subsequently measured as a surrogate for the core body temperature (*t*_*cr*_). At the end of the experiment, thermal images were taken via an infrared thermography (IRT) camera to predict the clothing temperature (*t*_*cl*_) and different local *t*_*sk*_ values at the face and hand regions.

### Environmental measurement

**Fig. 1:**
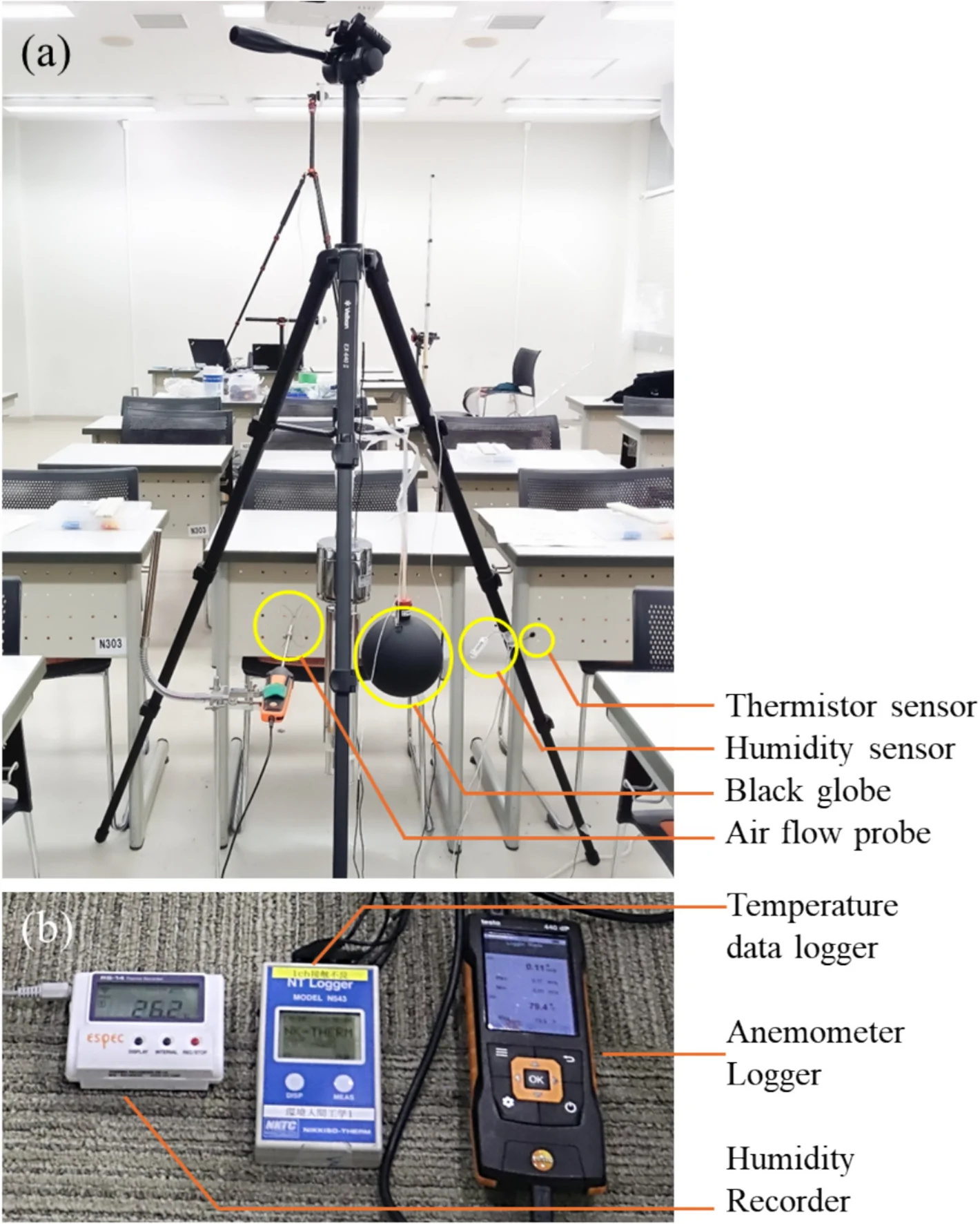
Data logger and sensor’s arrangement for the environmental data collection (**a**) and data loggers (**b**)

Four fundamental environmental factors related to the thermal environment *t*_*a*_, globe temperature (*t*_*g*_), *RH*, and *ν*_a_ were recorded during the experimental period. The data were collected at 1-s intervals. The data logger setup with the sensors is shown in Fig. 1. Three sets of data loggers were used to collect the environmental data from three different zones. Considering the center of an adult human body in a sitting posture, all the environmental data were recorded at a height of 0.6 m. During the survey, *t*_*a*_ was measured by using a thermistor sensor and recorded using a data logger (NR543R, Nikkiso-Thermo, Japan). The thermistor sensor probe was hung in open air and connected to a logger to record the *t*_*a*_ data. To record *t*_*g*_, the same logger and thermistor were used with a Bernon-type 150-mm black globe (Sibata, Japan), and at the center of the globe, the thermistor was placed. The* RH* data were recorded with a humidity recorder (RS-14WB, Espec, Japan), with a humidity sensor (RSH-4010, Espec, Japan). For *ν*_*a*_, an anemometer logger (Testo 440, Testo, Germany) with an omnidirectional hot-wire air flow probe (TUC 0628 0152, Testo, Germany) was used to record the air velocity from every direction. The data loggers were switched on 30 min prior to the survey time. Once the experiment was completed, the data were saved as a digital file. From the *t*_*g*_, *t*_*a*_, and *ν*_*a*_ data, *t*_*r*_ was calculated via the following formula [47]:

1 $$t_{r} = ((t_{g} + 273.15)^{4} + ((1.1 \times 10^{8} \times \nu_{a}^{0.6})/(\varepsilon \times D^{0.4})) \times (t_{g}-t_{a}))^{0.25}-273.15 (^\circ \mathrm{C})$$

where

*t*_g_ = Globe temperature (°C)

*ν*_*a*_ = Air velocity (m/s)

*ε* = Emissivity of the globe (usually 1.00 for a black globe)

*D* = Diameter of the globe (m) (0.15 m)

*t*_*a*_ = Air temperature (°C)

For individual *PMV* prediction, the values of *t*_*a*_, *t*_*r*_, *RH*, and *ν*_a_ measured at their working area were used for every participant.

### Questionnaire assessment

To identify individuals with cold constitution and non-cold constitution, a questionnaire survey was conducted. Table 3 shows the questionnaire used to assess the subjective symptoms of the cold constitution with the criteria for classifying individuals as having a cold constitution or non-cold constitution. This questionnaire was based on previous research on cold constitutions originally designed by Terasawa [34] and modified by Sakaguchi [33], which included some questions that were not used for classifying cold constitutions. These unused questions were excluded from the survey questionnaire used in this study (Table 3). From the survey, two groups were named as the CC group and the NC group.

**Table 3:** Questionnaire on cold constitution. This table was modified from a previous study [48]

Do you or are you_		
Category “a”	1	Compared to others, I tend to be more sensitive to the cold
	2	I suffer because my back, hands and feet, or some part of my body is cold
	3	I suffer in air-conditioned rooms because my body feels cold
	4	Compared to others, my hands and feet are cold
	5	Even in summer, my hands get cold
	6	Especially in winter, I sometimes cannot sleep because my feet are cold
Category “b”	7	I sometimes suffer because my entire body is cold
	8	I wear thick socks even in summer because my feet are cold
	9	Due to cold weather in winter, I always use an electric blanket, mattress, or pocket warmer
	10	I have been suffering from the cold for the last several years
	11	In winter and on cold days, I urinate more frequently
	12	Compared to others, my face is more pale
	13	My hands and feet always feel cold

Criteria for cold constitution: Answered yes, more than 2 in Category “a” questions. Answered yes, 1 in Category “a” question + more than 2 in Category “b” questions

To investigate the individual’s thermal assessment, an additional questionnaire survey was conducted on the thermal sensation vote (TSV), TC, TP, and DL according to ISO 28802 [12] guidelines. Participants were asked to vote TSV on a 7-point scale, where −3 to 3 correspond to cold, cool, slightly cool, neutral, slightly warm, warm, and hot in sequence. Additionally, TC (−3 to +3 characterized as very uncomfortable, uncomfortable, slightly uncomfortable, neither, slightly comfortable, comfortable, very comfortable), TP (−3 to 3 characterized as much cooler, cooler, slightly cooler, no change, slightly warmer, warmer, much warmer), and DL (1 to 4 characterized as satisfied, slightly satisfied, slightly non-satisfied, non-satisfied) were asked to evaluate the difference in thermal perceptions between the CC group and the NC group. For a clear understanding of the questionnaire, both the cold constitution questionnaire and the individual’s thermal assessment questionnaire were translated into Japanese according to their original meanings.

### PMV and PPD prediction

*PMV* is calculated by the following formula according to ISO 7730 [20]:

2 $$PMV = [0.303 \times exp(-0.036 \times M) + 0.028] \times \{(M-W)-3.05 \times 10^{-3} \times [5733-6.99 \times (M-W)-p_{a}]-0.42 \times [(M-W)-58.15]-1.7 \times 10^{-5} \times M \times (5867-p_{a})-0.0014 \times M \times (34-t_{a})-3.96 \times 10^{-8} \times f_{cl} \times [(t_{cl} + 273)4-(t_{r} + 273)4]-f_{cl} \times h_{c} \times (t_{cl}-t_{a})\}$$

where

*M* = Metabolic rate of human body [W/m^2^]

*W* = Rate of mechanical work [W/m^2^]

*p*_*a*_ = Water vapor pressure in ambient air [P_a_]

*t*_*a*_ = Air temperature [°C]

*f*_*cl*_ = Clothing area factor

*t*_*cl*_ = Mean temperature of the outer surface of the clothed body [°C]

*t*_*r*_ = Mean radiant temperature [°C]

*h*_*c*_ = Convective heat transfer coefficient [W/(m^2^∙K)]

Additionally, *PPD* was calculated based on *PMV* predictions.

3 $$PPD = 100-95 \times exp(-0.03353 \times PM{V}^{4}-0.2179 \times PM{V}^{2}) [{\%}]$$

### M prediction

In this study, to predict* M*, a method was adopted on the basis of the basal metabolic rate (*BMR*), physical activity ratio *(PAR)*, and body surface area *(BSA)* [49]. The *BMR* indicates the energy required to maintain vital activity in the waking state, in the supine position, and in a fasting situation (without breakfast) [50]. *PAR* is the ratio of the metabolic rate during specific activity to the *BMR* in unit time [51, 52]. Here,* M* was calculated by the following equation:

4 $$M = (BMR \times PAR)/BSA [\mathrm{W}/\mathrm{m}^{2}]$$

5 $$BMR = \{0.0481 \times W + 2.34 \times H-0.0138 \times age-0.4235 \times Gender\} \times 11.574 [\mathrm{W}]$$

From Fujimoto et al. [53], BSA was calculated by the following equation:

6 $$BSA = 0.008883 \times {W}^{0.444} \times (H \times 100{)}^{0.663} [{\mathrm{m}}^{2}]$$

where

*W* = weight [kg]

*H* = height [m]

*Age* = year

*Gender* = 1 for males and 2 for females

Here, the *PAR* for desk work was determined based on previous studies [52]. For males, *PAR* values of 1.35, 1.32, and 1.38 were considered for individuals with BMI < 18.5 kg/m^2^, 18.5 kg/m^2^ < BMI < 24.9 kg/m^2^, and BMI > 25 kg/m^2^, respectively, and for females, *PAR* values of 1.33, 1.34, and 1.36 were considered for individuals with a BMI < 18.5 kg/m^2^, 18.5 kg/m^2^ < BMI < 24.9 kg/m^2^, and BMI > 25 kg/m^2^, respectively.

### I_cl_ prediction

In this experiment, IRT was used to predict *t*_*cl*_, *t*_*sk*_, and *I*_*cl*_. To predict *I*_*cl*_, the following formula was used based on ISO 9920 [54],

7 $${I}_{cl} = ({t}_{sk}-{t}_{cl})/H [^\circ \mathrm{C}/\mathrm{W}\cdot {\mathrm{m}}^{2}]$$

8 $$\text{where }H = ({t}_{cl}-{t}_{o}) \times ({h}_{c} + {h}_{r})$$

Here, *t*_*o*_ = operative temperature [°C], where

9 $${t}_{o} = ({h}_{c} \times {t}_{a} + {h}_{r} \times {t}_{r})/({h}_{c} + {h}_{r})$$

*H* = Dry heat loss [W/m^2^]

*h*_*c*_ = Convective heat transfer coefficient [W/(m^2^∙K)]

*h*_*r*_ = Radiative heat transfer coefficient [W/(m^2^∙K)]

**Fig. 2:**
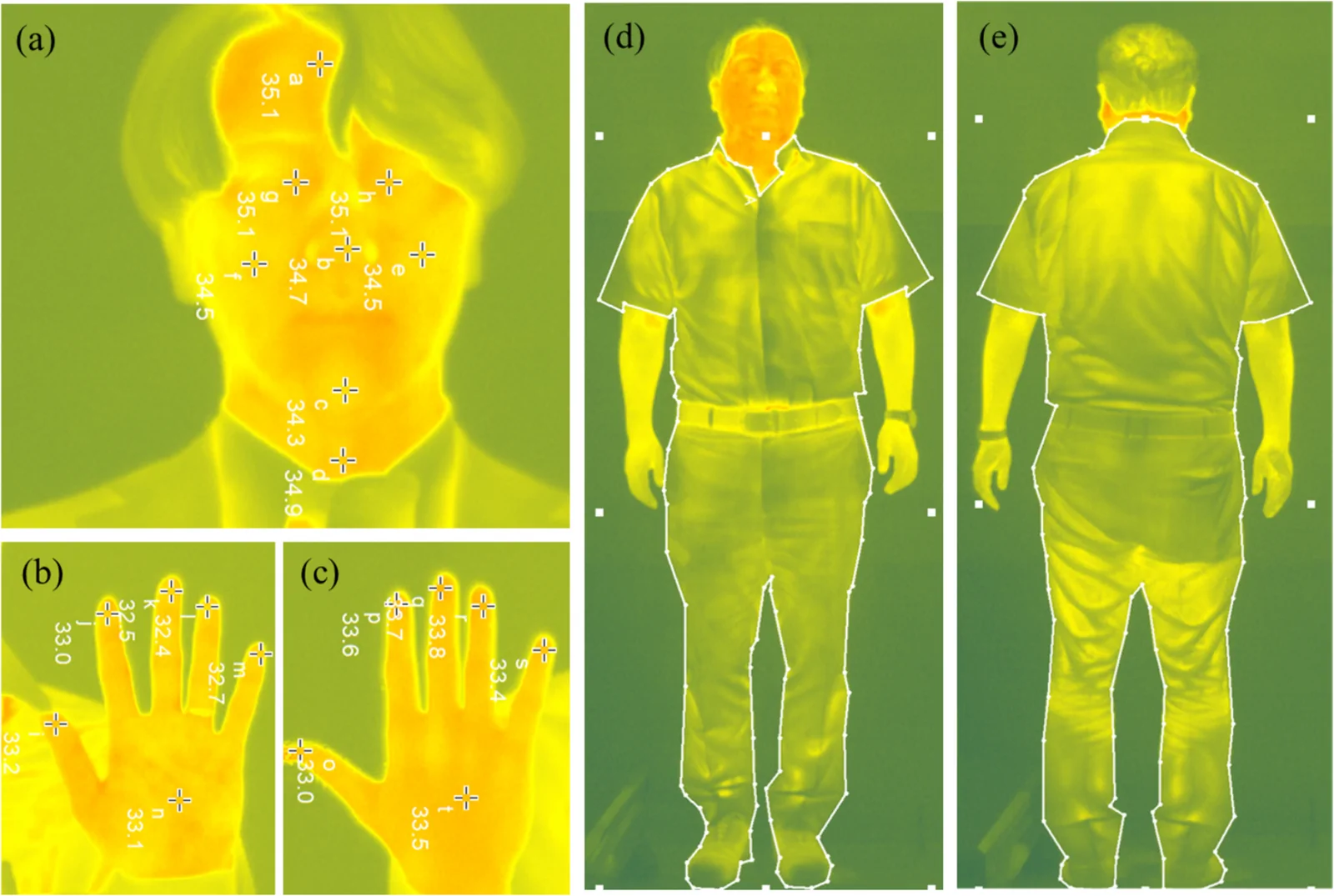
Thermal images for predicting the local skin temperature at the head region (**a**), palmar side of the hand (**b**), dorsal side of the hand (**c**), and clothing temperature prediction at the front side (**d**) and back side (**e**)

To predict *t*_*cl*_* and t*_*sk*_, thermal imaging was conducted using an IRT camera (R500EX-S, Nippon Avio, Japan). Figure 2 shows some samples of IRT images. The camera was positioned at a height of 0.90 m, ensuring that its plane was perpendicular to the body plane at the center of the body, considering the standing position [55]. The camera was turned on 30 min before the thermal photography for sensor stabilization. To predict *t*_cl_, from a distance of 4 m, front and backside images were taken, while the camera display was fully covered by the image of the whole body. To identify the facial and hand temperatures, images were taken from a distance of 1.5 m, while the camera display was covered by the face and hand segments, which were the regions of interest. For *t*_*cl*_ prediction, the average temperature of the clothing surface from the front and backside images was predicted by the camera manufacturer’s computer application (InfReC Analyzer NS9500 standard). Spot analysis was performed in the same application for predicting local *t*_*sk*_, where a hot spot was identified as a local *t*_*sk*_ for that particular zone. Data collection and processing were done following the guidelines outlined in the Thermographic Imaging in Sports and Exercise Medicine (TISEM) [56].

Considering the availability of an uncovered body surface appropriate for IRT imaging, the average temperature of the forehead (*t*_*forehead*_) and the center point of the dorsal hand (*t*_*dorsal hand*_) was calculated as surrogate measures for the mean *t*_*sk*_.

10 $${t}_{sk} = ({t}_{forehead} + {t}_{dorsal hand})/2 [^\circ \mathrm{C}]$$

The forehead near the body’s core mostly maintains a high skin temperature, while a distal extremity like the dorsal hand exhibits lower temperatures due to thermal environmental effects and vasomotor control. These concerns guided their selection for mean skin temperature assessment.

Additionally, for the validation of IRT temperature prediction, prior to the experiment, the IRT camera was calibrated by a true known temperature. The IRT camera was calibrated in a thermally insulated, dark climatic chamber maintained at a thermoneutral temperature of 25 °C. A Peltier module was covered with black tape (emissivity = 1.00) and served as a black body. It was heated with known temperatures ranging from 15 to 35 °C in increments of 5 °C. The actual temperatures were measured using a thermistor sensor connected to a data logger (NR543R, Nikkiso-Thermo, Japan). A regression analysis was performed to examine the correlation between the real temperatures and the IRT predicted temperatures, and a correction formula was applied to enhance the accuracy of the IRT measurements.

### Local t_sk_ and Δt_sk_ prediction

In the facial region, temperatures were measured via IRT at the forehead, nose, chin, neck, cheeks (averaged for both sides), and eye canthus (averaged for both eyes) through spot analysis, and the predicted temperatures were named as *t*_*forehead*_, *t*_*nose*_, *t*_*chin*_, *t*_*neck*_, *t*_*cheek*_, and *t*_*eye canthus*_, respectively. In the hand region, the dorsal and palmar surfaces, including the fingertips, were analyzed using the same method, and the predicted temperatures were named as *t*_*dorsal*_, *t*_*palm*_, *t*_*dorsal finger*_, and *t*_*palmar finger*_, respectively. Additionally, *Δt*_*forehead–nose*_ and *Δt*_*palm–palmar finger*_ were calculated as parameters reflecting the magnitude of vasoconstriction [57] to examine the association between Δ*t*_*proximal–distal*_ in individuals with a cold constitution.

### Statistical analysis

The differences in the mean values of TSV, TC, TP, and DL between the CC group and the NC group were analyzed using the Mann-Whitney *U*-test. The differences in *I*_*cl*_, *M*, *PMV*, *PPD*, local *t*_*sk*_, and *Δt*_*proximal–distal*_ between the two groups were analyzed by the non-paired Student’s *t*-test. Correlations between cold constitution (binary classification) and other factors were analyzed by Spearman’s rank correlation test. In the statistical analysis, cold constitution was represented as a dummy variable for statistical modeling. Here, cold constitution individuals were coded as 1, and non-cold constitution individuals were coded as 0. Additionally, gender was also encoded as a binary variable, where males were assigned a value of 0 and females were assigned a value of 1. This coding scheme was used to facilitate correlation analysis and other statistical procedures that require numerical input. All the data are presented as mean ± SD. In this study, a *p*-value threshold of 0.05 was used to determine statistical significance. All the analysis was performed using IBM SPSS Statistics version 20.

## Results

It was observed that in the NC group, the number of females was less compared with males. Additionally, in the NC group, body height, weight, and *BMI* were significantly higher compared with the CC group (*p* < 0.05, Table 1).

Table 2 illustrates the environmental conditions during the experiment in the office. It remained mostly stable and in a thermoneutral range throughout the experiment, both in summer and winter [19].

### Comparison of thermal sensations

**Fig. 3:**
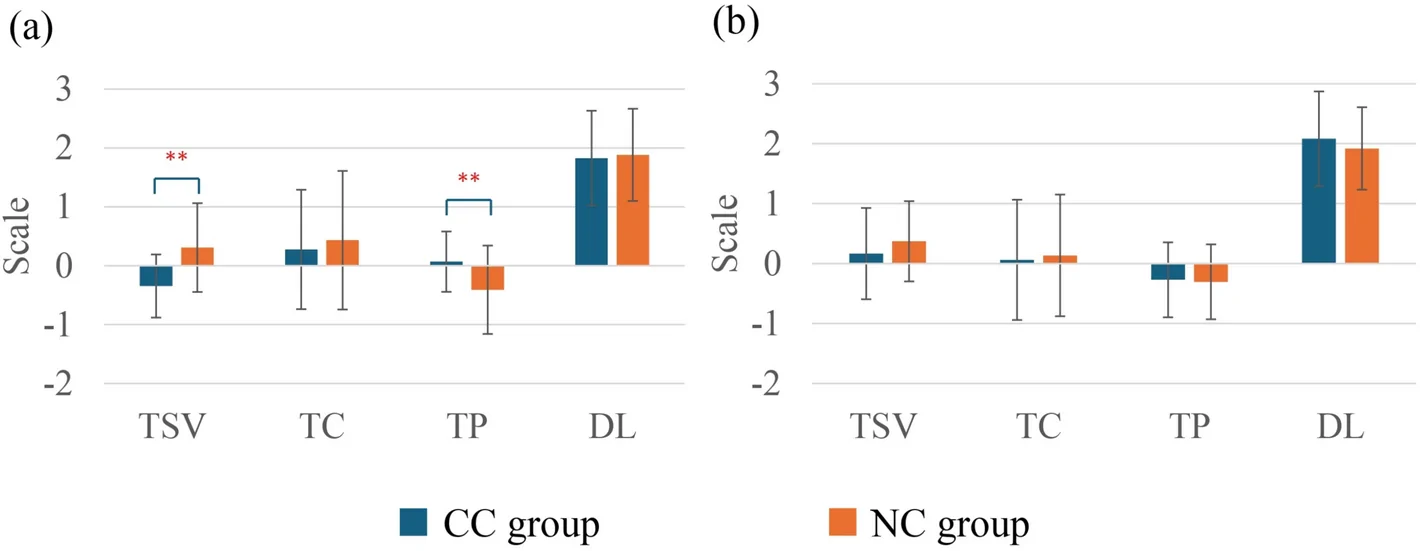
Comparison of thermal sensations between the CC group and the NC group in summer (**a**) and winter (**b**). In summer, CC group (*n* = 29) and NC group (*n* = 60); in winter, CC group (*n* = 24) and NC group (*n* = 51). Values are mean ± standard deviation. **p < 0.01 between the groups

Figure 3 illustrates the TSV, TC, TP, and DL comparisons between the CC group and the NC group. In summer, the CC group showed significantly lower TSV compared with the NC group (*p* < 0.01). Additionally, the CC group showed significantly higher TP compared with the NC group (*p* < 0.01). No significant differences were observed for TC and TP between the CC group and the NC group in summer. On the other hand, in winter, no significant differences were found in TSV, TC, TP, or DL between the two groups.

### Comparison of PMV and PPD

**Fig. 4:**
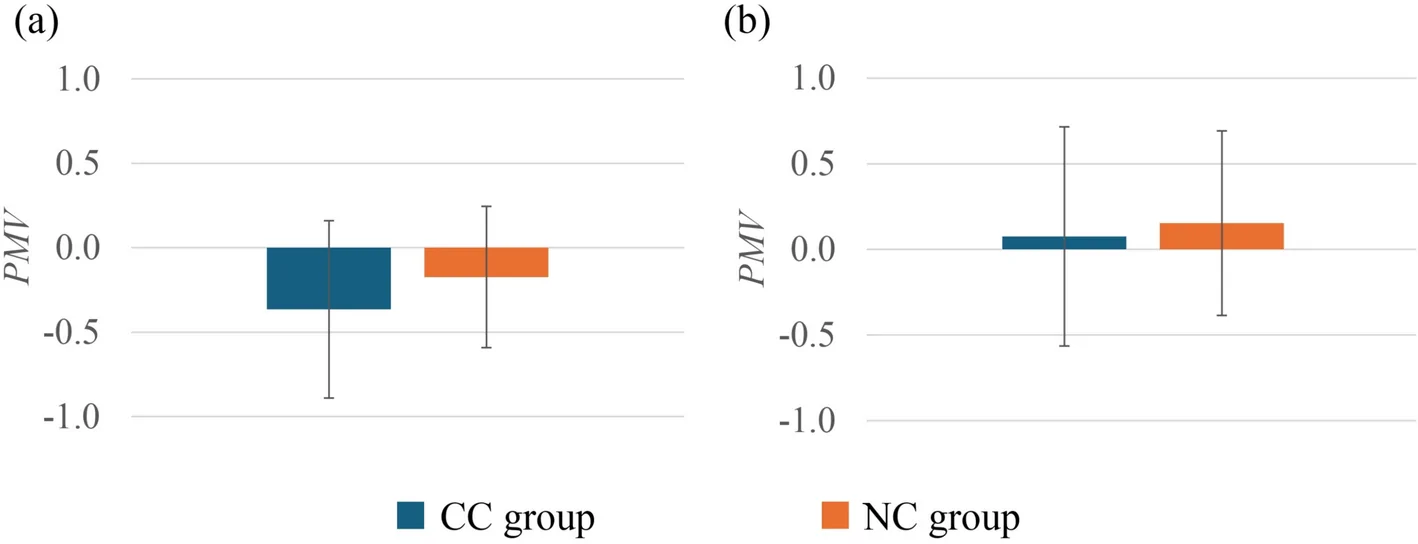
Comparison of *PMV* between the CC group and the NC group in summer (**a**) and winter (**b**). In summer, CC group (*n* = 29) and NC group (*n* = 60); in winter, CC group (*n* = 24) and NC group (*n* = 51). Values are mean ± standard deviation

**Fig. 5:**
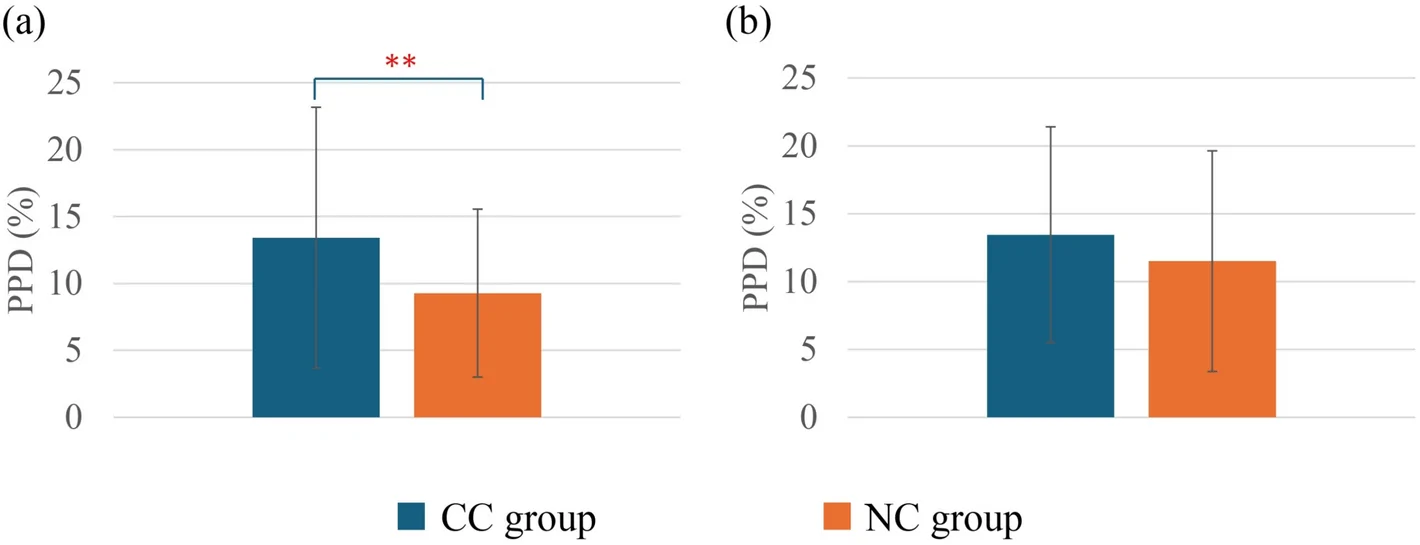
Comparison of *PPD* between the CC group and the NC group in summer (**a**) and winter (**b**). In summer, CC group (*n* = 29) and NC group (*n* = 60); in winter, CC group (*n* = 24) and NC group (*n* = 51). Values are mean ± standard deviation. **p < 0.01 between the groups

As shown in Fig. 4, there were no significant differences observed in *PMV* between the CC group and the NC group in either summer or winter. Figure 5 presents *PPD* comparison between the two groups. In summer, the CC group showed significantly higher *PPD* compared with the NC group (*p* < 0.05). However, in winter, there were no significant differences observed for *PPD* between the groups.

### Comparison of M

**Fig. 6:**
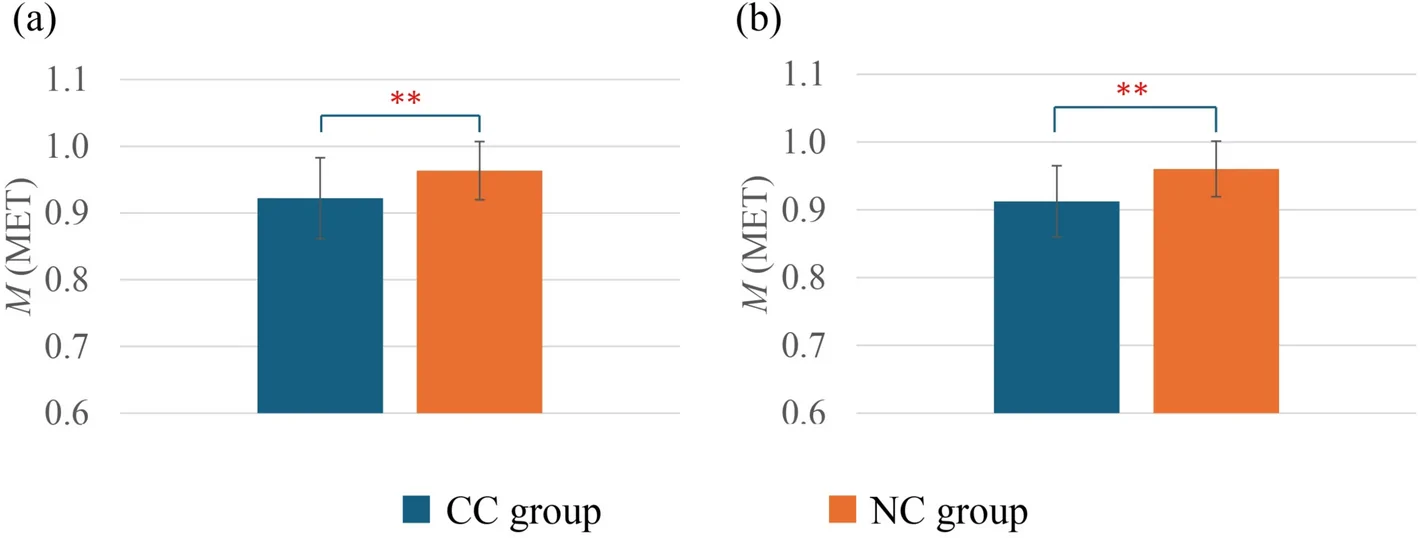
Comparison of *M* between the CC group and the NC group in summer (**a**) and winter (**b**). In summer, CC group (*n* = 29) and NC group (*n* = 60); in winter, CC group (*n* = 24) and NC group (*n* = 51). Values are mean ± standard deviation. **p < 0.01 between the groups

Figure 6 highlights the *M* comparison between the groups. The results revealed that the CC group had a significantly lower *M* in both seasons compared with the NC group.

### Comparison of I_cl_

**Fig. 7:**
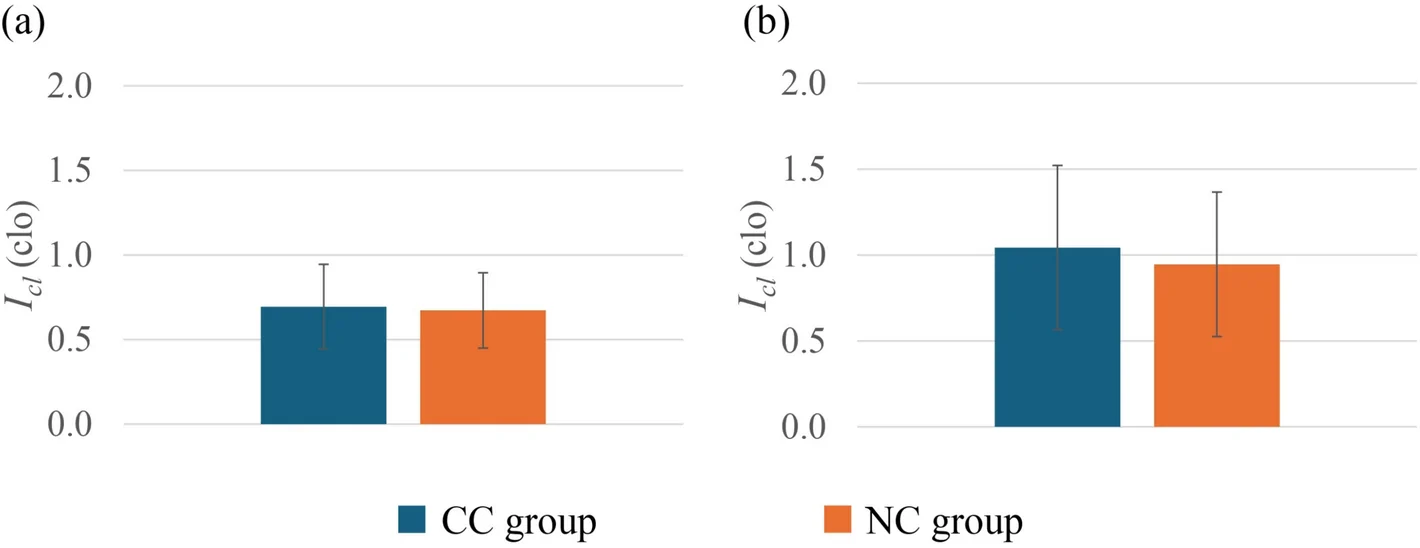
Comparison of *I*_*cl*_ between the CC group and the NC group in summer (**a**) and winter (**b**). In summer, CC group (*n* = 29) and NC group (*n* = 60); in winter, CC group (*n* = 24) and NC group (*n* = 51). Values are mean ± standard deviation

Figure 7 shows the *I*_*cl*_ comparison between the groups. No significant differences were observed for the *I*_*cl*_ between the groups in either summer or winter. However, when the summer *I*_*cl*_ and the winter *I*_*cl*_ were compared, the *I*_cl_ in winter was significantly greater than that in the summer for both the groups (*p* < 0.01)

### Comparison of local t_sk_ and Δt_sk_

**Fig. 8:**
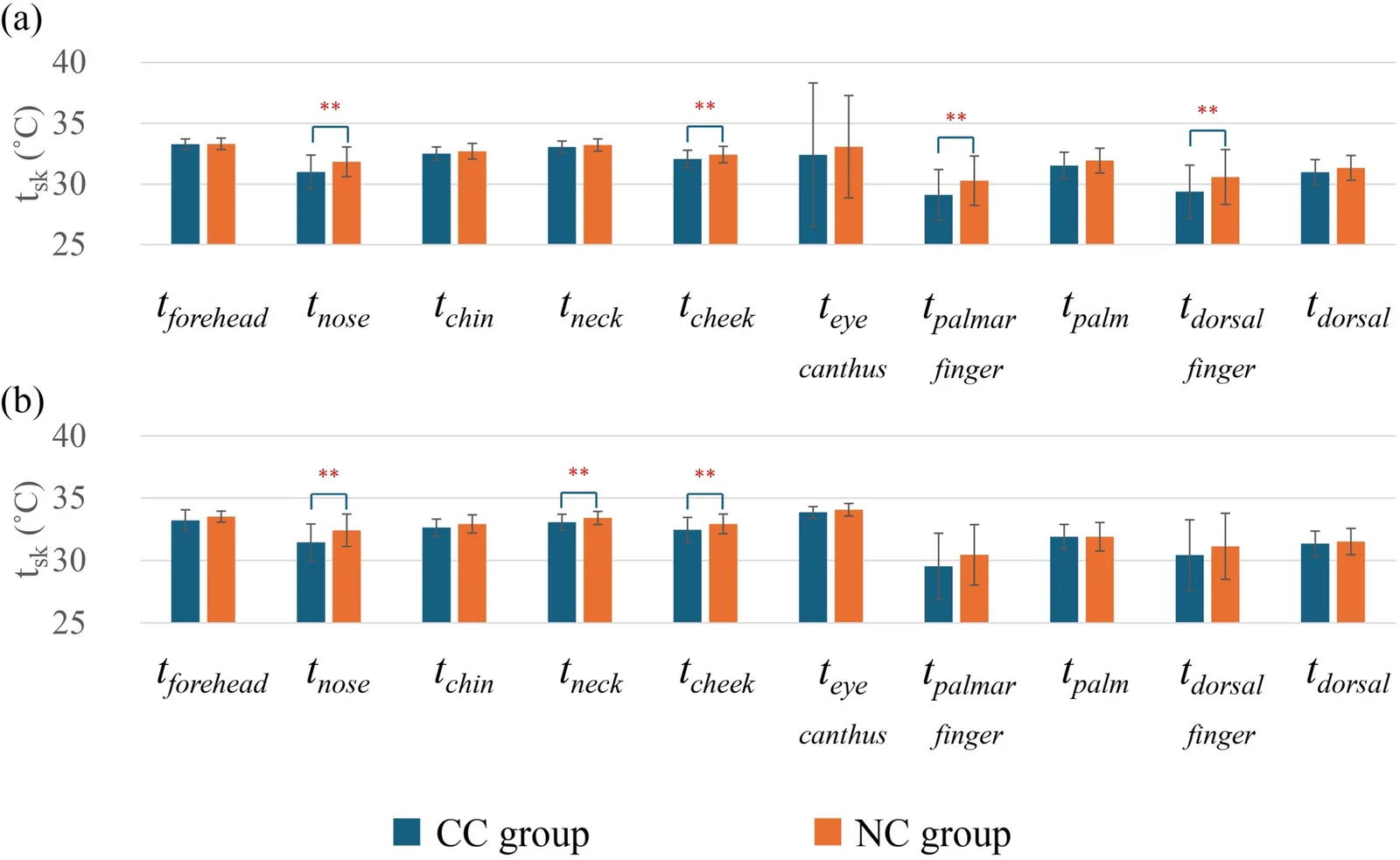
Comparison of local *t*_*sk*_ between the CC group and the NC group in summer (**a**) and winter (**b**). In summer, CC group (*n* = 29) and NC group (*n* = 60); in winter, CC group (*n* = 24) and NC group (*n* = 51). Values are mean ± standard deviation. **p < 0.01 between groups

**Fig. 9:**
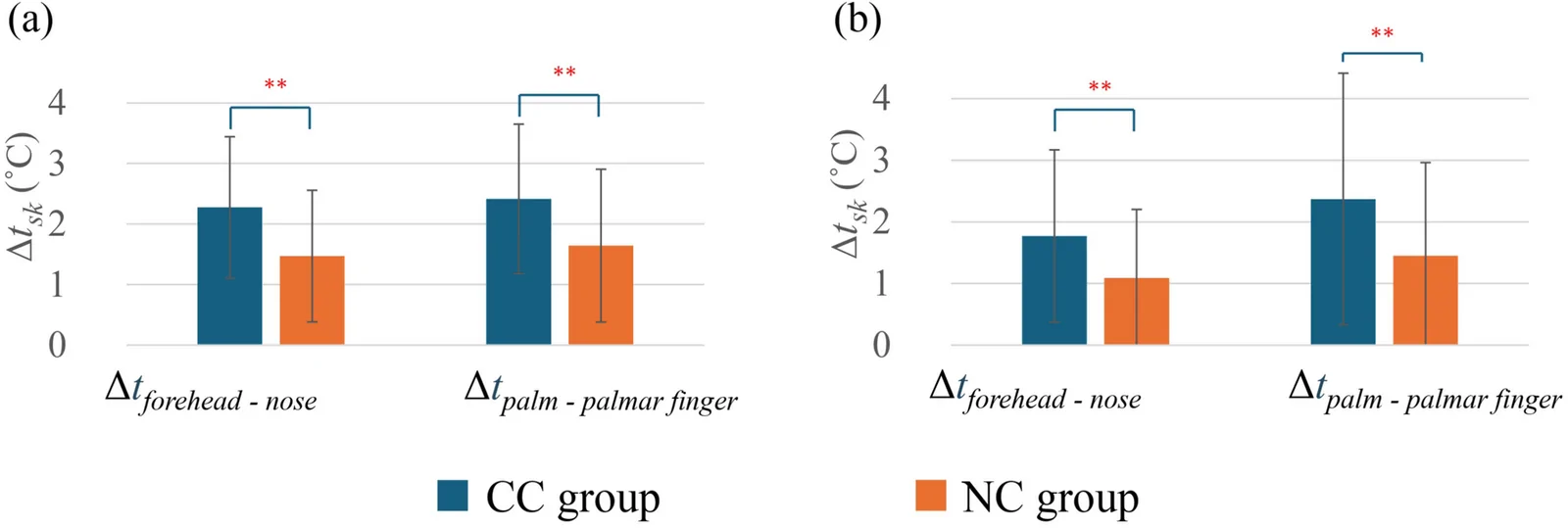
Comparison of *Δt*_*sk*_ between the CC group and the NC group in summer (**a**) and winter (**b**). In summer, CC group (*n* = 29) and NC group (*n* = 60); in winter, CC group (*n* = 24) and NC group (*n* = 51). Values are mean ± standard deviation. **p < 0.01 between the groups

Figure 8 represents the local skin temperature comparison between the groups. It was observed that the CC group has relatively lower skin temperature compared with the NC group. Especially in the summer, the CC group had significantly lower *t*_*nose*_, *t*_*cheek*_, *t*_*palm*_, and *t*_*dorsal*_ compared with the NC group (*p* < 0.05). In the winter, the CC group also showed significantly lower *t*_*nose*_, *t*_*neck*_, and *t*_*cheek*_ compared with the NC group (*p* < 0.05). Figure 9 illustrates the temperature difference between *t*_*forehead*_ and *t*_*nose*_, as well as *t*_*palm*_ and *t*_*palmar finger*_. The CC group had significantly greater *Δt*_*forehead–nose*_ and *Δt*_*palm–palmar finger*_ compared with the NC group both in summer (*p* < 0.05) and winter (*p* < 0.01).

### Correlation of gender, BMI, I_cl_, M, TSV, TC, TP, DL, PMV, and PPD with cold constitution

Table 4 represents the relationships between cold constitution and various factors, including gender, *BMI*, *I*_*cl*_, *M*, TSV, TC, TP, DL, *PMV*, and *PPD*. Additionally, the partial correlations among these factors were analyzed to examine their relationships with the cold constitution, and the results are shown in Table 4. In summer, gender, *BMI*, *M*, TSV, TP, *PMV*, and *PPD* had significant moderate correlations with the cold constitution (*ρ* = 0.33, −0.38, −0.33, −0.44, 0.30, −0.21, and 0.27, respectively, *p* < 0.05 for each). In addition, in partial correlation analysis, when controlling for the gender factor, correlation of the *BMI* and the cold constitution became weak, although it was significant (*ρ* = −0.27, *p* < 0.05). The correlation with TSV remains significant (*ρ* = −0.35, *p* < 0.01), and TP remains a moderate positive correlation (*ρ* = 0.27, *p* < 0.05). When controlling for *BMI* in the partial correlation analysis, only TSV and TP were significantly correlated with cold constitution (*ρ* = −0.31 and −0.21, respectively, *p* < 0.05). When controlling for *I*_*cl*_, significant correlations with the cold constitution were observed for gender, *BMI*, *M*, TSV, TP, *PMV*, and *PPD* (*ρ* = 0.30, −0.37, −0.34, −0.38, 0.29, −0.24, and 0.30, respectively, *p* < 0.01, for gender, *BMI*, *M*, TSV, TP, and *PPD*, *p* < 0.05 for *PMV*). When *M* was controlled, the correlation weakened and only for TSV and TP were significantly correlated with cold constitution (*ρ* = −0.31 and 0.21 respectively, *p* < 0.05 for each). When gender and *BMI* were controlled together, most of the correlation disappeared, even though TSV and TP were still significantly correlated with the cold constitution (*ρ* = −0.31 and 0.22, respectively, *p* < 0.05 for each).

**Table 4:** Correlations of cold constitution with gender, *BMI*, *I*_*cl*_, *M*, thermal perceptions, *PMV *and *PPD*

	Gender	*BMI*	*I*_*cl*_	*M*	TSV	TC	TP	DL	*PMV*	*PPD*
**Summer**										
*ρ*-value	0.33**	−0.38**	−0.01	−0.33**	−0.44**	−0.10	0.30**	−0.03	−0.21*	0.27*
Partial ρ (controlled: gender)	_	−0.27*	0.13	−0.18	−0.35**	−0.13	0.27*	−0.07	−0.20	0.20
Partial ρ (controlled: *BMI*)	0.16	_	0.13	−0.16	−0.31*	−0.20	0.21*	0.02	−0.14	0.20
Partial ρ (controlled: *I*_*cl*_)	0.30**	−0.37**	_	−0.34*	−0.38**	−0.12	0.29**	−0.00	−0.24*	0.30**
Partial ρ (controlled: *M*)	0.05	−0.21	0.12	_	−0.31*	−0.17	0.21*	−0.04	−0.18	0.14
Partial ρ (controlled: gender, *BMI*)	_	_	−0.06	−0.04	−0.31**	−0.14	0.22*	−0.06	−0.11	0.16
**Winter**										
*ρ*-value	0.48**	−0.42**	0.13	−0.39**	−0.10	0.01	0.02	0.09	−0.04	0.10
Partial ρ (controlled: gender)	_	−0.29*	−0.15	−0.14	−0.22	−0.05	0.01	0.07	−0.15	−0.09
Partial ρ (controlled: *BMI*)	0.37**	_	0.10	−0.29*	−0.04	−0.03	−0.03	0.10	−0.01	0.10
Partial ρ (controlled: *I*_*cl*_)	0.49**	−0.42**	_	−0.44**	−0.15	−0.04	0.02	0.09	−0.32**	0.06
Partial ρ (controlled: *M*)	0.23*	−0.23*	−0.03	_	−0.14	−0.11	−0.06	0.17	−0.03	0.04
Partial ρ (controlled: gender, *BMI*)	_	_	−0.10	−0.02	−0.14	−0.04	−0.03	0.08	−0.10	−0.06

Values are correlation coefficient (*ρ*-value)

*BMI* body mass index, *I*_*cl*_ clothing insulation, *M* metabolic rate, *TSV* thermal sensation vote, *TC* thermal comfort, *TP* thermal preference, *DL* dissatisfaction level, *PMV* predicted mean vote, *PPD* predicted percentage of dissatisfied

*Significant correlation (*p* < 0.05)

**Significant correlation (*p* < 0.01)

In winter gender, *BMI* and *M* showed significant correlation with cold constitution (*ρ* = 0.48, −0.42, and −0.39, respectively, *p* < 0.01 for each). In partial correlation, controlling for gender weakened the correlation, and only *BMI* was significantly correlated with cold constitution (*ρ* = −0.29, *p* < 0.05). In the *BMI*-controlled correlation analysis, gender and *M* still showed significant but weaker correlations (*ρ* = 0.37 and −0.29, respectively, *p* < 0.01 for gender, *p* < 0.05 for M). Controlling *I*_*cl*_ had little impact on most of the correlations, and gender, *BMI*, *M*, and *PMV* were significantly correlated (*ρ* = 0.49, −0.42, −0.44, and −0.32, respectively, *p* < 0.01 for each). When controlling for *M*, most correlations weakened, although gender and *BMI* were significantly correlated (*ρ* = 0.23 and −0.23, respectively, *p* < 0.01 for gender, *p* < 0.05 for *BMI*). When gender and *BMI* were controlled together, the correlation disappeared.

### Correlation of t_sk_ and Δt_proximal–distal_ with cold constitution

In Table 5, the correlations between cold constitution and local *t*_*sk*_ as well as *Δt*_*proximal–distal*_ are summarized. In summer, several significant correlations were noted. Significant negative correlations were figured out for *t*_*nose*_, *t*_*cheek*_, *t*_*palm*_, and *t*_*dorsal finger*_ with cold constitution (*ρ* = −0.27, −0.21, −0.27, and −0.18 respectively, *p* < 0.05 for each). Significant positive correlations were observed for *Δt*_*forehead–nose*_ and *Δt*_*palm–palmar finger*_ with the cold constitution (*ρ* = 0.31 and 0.30, respectively, *p* < 0.05 for each). In the partial correlation analysis while controlling for gender, *BMI*, and *M*, the magnitude of most of the correlations reduced. However, *Δt*_*forehead–nose*_ was significantly correlated with the cold constitution, while gender and *M* were controlled (*ρ* = 0.24 and 0.23, respectively, *p* < 0.05 for each). Whereas when the *I*_*cl*_ was controlled, most of the correlation elevated slightly, and the *t*_*nose*_, *t*_*cheek*_, *t*_*palmar finger*_, and *t*_*dorsal finger*_ were significantly negatively correlated with the cold constitution (*ρ* = −0.33, 0.23, −0.25, and −0.25, respectively, *p* < 0.05 for each). Additionally, positive correlations were identified for *Δt*_*forehead–nose*_ and *Δt*_*palm–palmar finger*_ (*ρ* = 0.37 and 0.27, respectively, *p* < 0.01 for *Δt*_*forehead–nose*_ and *p* < 0.05 for *Δt*_*palm–palmar finger*_). While gender and *BMI* were controlled together, no significant correlation was observed with cold constitution.

**Table 5:** Correlation of the cold constitution with local *t*_sk_ and *t*_sk_ differences

	*t*_*forehead*_	*t*_*nose*_	*t*_*chin*_	*t*_*neck*_	*t*_*cheek*_	*t*_*eye canthus*_	*t*_*palmar finger*_	*t*_*palm*_	*t*_*dorsal finger*_	*t*_*dorsal*_	*Δt*_*forehead*__–__*nose*_	*Δt*_*palm*__–__*palmar finger*_
**Summer**												
*ρ*-value	−0.08	−0.27*	−0.14	−0.15	−0.21*	−0.17	−0.17	−0.27*	−0.18*	−0.26*	0.31**	0.30**
Partial ρ (controlled: gender)	0.03	−0.21	−0.09	−0.12	−0.14	−0.09	−0.15	−0.11	−0.14	−0.10	0.24*	0.16
Partial ρ (controlled: *BMI*)	−0.03	−0.19	−0.08	−0.15	−0.17	−0.06	−0.13	−0.09	−0.10	−0.08	0.21	0.13
Partial ρ (controlled: *I*_*cl*_)	−0.01	−0.33*	−0.16	−0.13	−0.23*	−0.06	−0.25*	−0.17	−0.25*	−0.15	0.37**	0.27*
Partial ρ (controlled: *M*)	0.04	−0.19	−0.10	−0.12	−0.14	−0.02	−0.16	−0.12	−0.15	−0.12	0.23*	0.16
Partial ρ (controlled: gender, *BMI*)	−0.00	−0.15	−0.07	−0.14	−0.13	−0.07	−0.09	−0.07	−0.06	−0.07	0.17	0.09
**Winter**												
*ρ*-value	−0.23*	−0.33**	−0.20	−0.27*	−0.23	−0.23*	−0.01	−0.17	−0.09	−0.13	0.25*	0.21
Partial ρ (controlled: gender)	−0.13	−0.16	−0.08	−0.19	−0.12	−0.15	−0.07	−0.00	−0.02	−0.11	0.12	0.10
Partial ρ (controlled: *BMI*)	−0.19	−0.25*	−0.14	−0.30*	−0.12	−0.16	−0.14	−0.06	−0.08	−0.09	0.19	0.17
Partial ρ (controlled: *I*_*cl*_)	−0.21	−0.31**	−0.17	−0.27*	−0.25*	−0.18	−0.16	0.02	−0.10	−0.08	0.25*	0.23*
Partial ρ (controlled: *M*)	−0.09	−0.17	−0.11	−0.21	−0.13	−0.14	−0.14	−0.07	−0.07	−0.14	0.15	0.16
Partial ρ (controlled: gender, *BMI*)	−0.11	−0.14	−0.06	−0.22	−0.05	−0.13	−0.07	−0.04	−0.01	−0.11	0.09	0.07

*BMI* body mass index, *I*_*cl*_ clothing insulation, *M* metabolic rate, *t*_*forehead*_ forehead skin temperature, *t*_*nose*_ nose skin temperature, *t*_*chin*_ chin skin temperature, *t*_*neck*_ neck skin temperature, *t*_*cheek*_ cheek skin temperature, *t*_*eye canthus*_ eye canthus skin temperature, *t*_*dorsal*_ dorsal hand skin temperature, *t*_*palm*_ palmar hand skin temperature, *t*_*dorsal finger*_ dorsal finger skin temperature, and *t*_*palmar finger*_ palmar finger skin temperature. Values are correlation coefficient (*ρ*-value)

*Significant correlation (*p* < 0.05)

**Significant correlation (*p* < 0.01)

In winter, significant negative correlations were recorded for *t*_*forehead*_, *t*_*nose*_, *t*_*neck*_, and *t*_*eyecanthus*_ (*ρ* = −0.23, −0.33, −0.27, and −0.23, respectively; *p* < 0.05 for *t*_*forehead*_, *t*_*neck*_, and *t*_*eyecanthus*_; *p* < 0.05 for *t*_*nose*_). A significant positive correlation was observed for *Δt*_*forehead–nose*_ with the cold constitution (*ρ* = 0.25, *p* < 0.05). Controlling for *BMI* reduced correlation levels, and statistical significance was noted for *t*_*nose*_ and *t*_*neck*_ (*ρ* = −0.25 and −0.30, respectively, *p* < 0.05 for each). When controlling for gender, *M*, and gender combined with *BMI*, no significant correlations were observed. However, controlling for *I*_*cl*_, the correlations slightly increased, and significant negative correlations were retained for *t*_*nose*_, *t*_*neck*_, and *t*_*cheek*_ with cold constitution (*ρ* = −0.31, −0.27, and −0.25, respectively, *p* < 0.05 for *t*_*neck*_ and *t*_*cheek*_, *p* < 0.01 for *t*_*nose*_). Additionally, positive correlations were observed for *Δt*_*forehead–nose*_ and *Δt*_*palm–palmar finger*_ with the cold constitution (*ρ* = 0.25 and 0.23, respectively, *p* < 0.05 for each).

## Discussion

This research was conducted to investigate thermal perceptions, *PMV*, *PPD*, and physiological responses such as local skin temperatures in relation to the cold constitution. This study also explored the associations of morphological characteristics and personal factors influencing thermal perception with cold constitution to gain a more comprehensive understanding of those aspects related to cold constitution.

From the morphological characteristics, it was observed that mostly in the NC group, the female participants were fewer than males, which is consistent with the findings of some prior studies [58]. Notably, the body height, weight, and *BMI* of the individuals in the NC group were significantly higher than those in the CC group. This aligns with some previous research, where researchers also mentioned the association of thinness with cold constitution [59]. Previous research has shown that individuals with lower *BMI* have less insulation due to lower fat mass, and that individuals with lower *BMI* feel colder than those with higher *BMI* do [32, 60]. Possibly due to this leanness, cold constitution individuals tend to feel colder compared to others, and this aligns with the findings in this research on TSV, TC, TP, and DL differences between the CC group and the NC group (Fig. 3).

Based on the correlation analysis, it was observed that gender, *BMI*, and *M* were strongly significantly correlated with cold constitution in both summer and winter (Table 4). The strong positive correlations between gender and cold constitution suggest that females tend to display a higher cold constitution than males do. Even while controlling for the other factors in partial correlation analysis, gender remained a confounding determinant. This finding supports the existing research on physiological differences between genders, such as females having lower *BMI* and lower *M*, which influenced their cold thermal sensitivity. This finding is consistent with previous research [32, 58]. In both seasons, *BMI* consistently showed a moderate negative correlation with cold constitution, which reflected that individuals with lower *BMI* felt colder.

This experiment was conducted in a thermoneutral office environment both in the summer and the winter [12] (Table 2). However, the findings showed that in summer, TSV and TP had significant differences between the CC group and the NC group (Fig. 3). The CC group reported higher cold than did the NC group. Additionally, the CC group voted their thermal preference warmer compared to the NC group. This result supports the basic principle of cold constitution where cold constitution individuals feel colder than the other individuals do [61]. This finding also reinforces the fundamental concept of cold constitution. However, in winter, there were no significant differences observed between the groups. Considering the personal factors associated with human heat balance, *M* was quite similar across the two seasons for each group separately (Fig. 6); however, their *I*_cl_ was significantly higher in the winter than in the summer clothing insulation for both groups. Possibly, higher *I*_*cl*_ in winter was a factor which mitigated the thermal perception differences between the groups and elevated the thermal sensation, especially for the CC group. This may explain why both groups’ average TSV values were on the warmer side in the winter because of higher *I*_*cl*_. It seems that outdoor environmental conditions influenced individuals to wear more clothing during winter, even though the indoor conditions were mostly similar in summer and winter. Office norms can be another reason which influenced their wearing of clothing. In addition, some researchers have also mentioned the impact of adaptation on thermoregulatory responses and thermal sensations [62–64]. It is also possible that in this experiment, in the winter, a colder outdoor environment helped to adapt better in that thermoneutral office environment. This could also be another reason for elevated TSV for both groups.

Among the different thermal perceptions, TSV showed significant negative correlations in summer. Even in the partial correlation assessment, controlling for gender, *BMI*, *I*_*cl*_, *M*, or gender and *BMI* together, the correlation between TSV and cold constitution remained significant, suggesting that thermal sensation is strongly associated with cold constitution. Additionally, TP showed weak-to-moderate positive correlations even when controlling for several other parameters, which indicates a strong link between TP and the cold constitution.

However, there was no significant direct correlation observed between *I*_*cl*_ and the cold constitution, suggesting that *I*_*cl*_ was not significantly associated with the cold constitution. In addition, when *I*_*cl*_ was controlled for partial correlation, the general correlation values of gender, *BMI*, and *M* with cold constitution did not have a substantial effect, which also indicates the minimum influence of cold constitution on *I*_*cl*_. Controlling the *I*_*cl*_ is a type of behavioral thermoregulation. However, the results suggested that people with cold constitution did not have enough behavioral adaptations to cold. It was anticipated that individuals in the CC group, due to their heightened cold sensation, would exhibit higher clothing insulation levels. However, this trend was not observed, even though they reported cold sensation in summer. This suggests that clothing culture or social norms may have outweighed the influence of thermal sensation. Possibly, that is the reason for no significant correlation observed between cold constitution and *I*_*cl*_.

When *PMV*, which represents the mathematical prediction of thermal sensation, was investigated, no significant differences were observed between the groups (Fig. 4). Nevertheless, in both summer and winter, *PMV* predictions were quite relatable to TSV. Additionally, in summer, *PPD* predictions showed significant differences between the CC group and the NC group (Fig. 5). The CC group showed a higher dissatisfaction percentage compared to the NC group, which is relatable to the cold constitution principles. Even under thermoneutral conditions, cold constitution people feel cold and dissatisfied [65], whereas in winter possibly higher clothing insulation (Fig. 7) was the factor that moderated the *PPD* difference levels between the CC group and the NC group (Fig. 5). In the correlation analysis, *PMV* and *PPD* showed mostly weak but significant correlations with the cold constitution in summer. On the other hand, it is possibly because the higher *I*_*cl*_ in winter reduces heat loss from the body, buffering the effect of the cold constitution on thermal sensation. This *I*_*cl*_ could be the effect that diminishes the direct relationship between the cold constitution and TSV, TP, *PMV*, and *PPD* in winter.

In terms of metabolic heat production, it was observed that both in the summer and the winter, the CC group had significantly lower *M* compared to the NC group, which is consistent with previous studies [42]. This can be explained by the prominent presence of female individuals in the CC group as well as lower *BMI* individuals in the CC group (Table 1). Several studies focused on cold constitution have demonstrated the association of females and lower *BMI* with cold constitution [40, 58]. Gender and *BMI* are the two key parameters associated with *M*. In this study, the prominent presence of females in the CC group as well as lower *BMI* was the primary factors of lower *M*. This can also be related to the basic concept of human heat balance, where it can be argued that owing to a lower metabolic rate, individuals with cold constitution have reduced heat storage compared with those without cold constitution. Reduced heat storage occurs with a reduction in the mean body temperature. In this study, no difference in core body temperature was observed between the groups. Thus, the mean *t*_*sk*_ might be lower in the CC group. As a result, people with a cold constitution tend to feel colder than those without cold constitution. In addition, possibly that is why *M* showed a strong significant correlation with cold constitution both in summer and winter (Table 4). *M* also correlated significantly, even when controlling for other factors. This result suggested that individuals with lower metabolic rates generate less body heat and tend to have a cold constitution.

Another personal factor directly associated with thermal sensation is clothing insulation [20]. Significantly higher *I*_*cl*_ in the winter than in the summer for both groups demonstrates the behavioral adaptation of thermoregulation. It was observed that in summer, the CC group reported feeling significantly colder than the NC group did, despite both groups having nearly the same clothing insulation. Most likely, clothing culture and norms suppressed behavioral adaptation and likely led to wearing lighter clothing for the CC group in the summer, even though they felt cold. That can be the reason for the lack of significant correlation observed for *I*_*cl*_ with the cold constitution. However, in winter, higher clothing insulation enabled cold constitution individuals to adapt comfortably to the thermal environment.

According to the local skin temperature comparison between the CC group and the NC group, in most cases, the CC group had a lower local skin temperature. Specifically, *t*_*nose*_, *t*_*cheek*_, *t*_*palmar finger*_, and *t*_*dorsal finger*_ were significantly lower in the CC group compared to the NC group in summer (Fig. 8). For instance, some studies have shown that distal body parts are the earliest to feel cold [66], and individuals with cold constitution tend to have lower *t*_*sk*_ in those areas [28]. It can be described as the association of vasoconstriction with the cold constitution along with the tendency to perceive a thermoneutral environment as cold [40]. In a cold environment, the sympathetic nervous system releases noradrenaline (NA) from the nerve endings and binds to adrenergic receptors on the vascular smooth muscle cell membrane to induce vasoconstriction [67]. It has also been reported that long-term local cold exposure reduces endothelial nitric oxide (NO) synthase activity, which suppresses NO-mediated vasodilation [68]. It modulates vasoconstriction by activating intracellular signalling pathways that regulate smooth muscle contraction [68]. It reduces blood flow to the distal part of the body caused by vasoconstriction [69]. Possibly, that is the reason behind the significantly lower *t*_*nose*_, *t*_*cheek*_, *t*_*palmar finger*_, and *t*_*dorsal finger*_ in the CC group compared to the NC group. Additionally, the presence of high arteriovenous anastomoses (AVAs) in the nose and finger regions is also associated with the lower skin temperature [70, 71]. In those areas, vasoconstriction due to sympathetic activation appears to be more pronounced than in other skin regions, likely due to the higher presence of AVAs compared to other skin regions. Remarkably, in the winter, *t*_nose_, *t*_neck_, and *t*_cheek_ showed significantly lower values in the CC group than in the NC group. There were no significant differences observed in the hand region. One possible reason can be highlighted as higher *I*_*cl*_ in the winter than in the summer for both groups. Mostly it was observed that the extra clothing was used to cover the trunk body, and particularly in the winter, people tended to wear full-sleeve clothing. It is possible that this kind of clothing could increase the hand region temperature for people with cold constitution. However, in the face area, there was no effect of clothing. This may explain the consistent local skin temperature difference in the face region between the CC group and the NC group, even in winter. Therefore, it can be presumed that no correlation was found between *I*_*cl*_ and the cold constitution; however, a higher *I*_*cl*_ may serve as a behavioural adaptation to reduce body heat loss and enhance comfort for cold constitution people.

*Δt*_*forehead–nose*_ and *Δt*_*palm–palmar finger*_ were significantly higher for the CC group in both summer and winter (Fig. 9). This result clearly indicates that the vasoconstriction phenomenon occurred at the distal part of the body for the cold constitution participants to suppress heat dissipation from the skin surface. This event also matches the findings of previous studies [29, 68]. A lower skin temperature at the distal part of the body may lead to a cold sensation in individuals with a cold constitution.

In Table 5, the results demonstrate significant negative correlations between the cold constitution and local *t*_*sk*_ as well as positive correlations between the cold constitution and *Δt*_*proximal–distal*_. These results highlight the strong relationship between physiological responses and cold constitution, which has also been confirmed by prior research [44]. These findings indicate that individuals with cold constitution tend to exhibit lower skin temperatures, particularly in peripheral and exposed areas such as the nose, cheek, and fingers. In the partial correlation assessment, adjusting *I*_*cl*_ consistently strengthened the negative correlations between the cold constitution and local *t*_*sk*_, and this emphasized the role of *I*_*cl*_ in modulating local skin temperatures. Adjusting for gender or *BMI* or gender and *BMI* together reduced the strength of most correlations, suggesting that these factors may partially contribute to the correlation between the cold constitution and local *t*_*sk*_. Controlling for *M* also had a slight effect on the correlations, indicating that the association between cold constitution and local *t*_*sk*_ is slightly influenced by variations in *M*.

Furthermore, *Δt*_*proximal–distal*_ was positively correlated with the cold constitution. This suggests that individuals with a cold constitution tend to have greater temperature differences between the proximal and distal regions, potentially reflecting impaired thermoregulation or reduced peripheral blood flow, which represents the association of vasoconstriction with the cold constitution [45]. Partial correlations revealed that controlling for *I*_*cl*_ generally enhanced the correlations between *Δt*_*proximal–distal*_ and the cold constitution, which indicates that *I*_*cl*_ also plays a critical role in moderating regional thermal differences.

## Conclusion

This study aimed to explore the associations of thermal perceptions, *PMV*, *PPD*, and physiological responses such as local skin temperatures in relation to cold constitution. It also inspected the differences between the CC group and the NC group in those aspects. The following conclusions can be drawn from the research findings:

1. Cold constitution survey, correlation analysis, and partial correlation analysis identified gender and *BMI* as key predictors of cold constitution. Compared with males, females presented a greater prevalence of cold constitution, whereas an increased BMI reduced the likelihood of cold constitution.
2. CC group felt colder and preferred warmer environments than did the NC group in summer, even after adjusting for gender and *BMI*, whereas in winter thermal sensation and preference showed no significant differences, though *I*_*cl*_ was significantly higher in winter for both groups.
3. *PMV* showed no significant group differences, but in summer, the CC group’s *PPD* was significantly higher than the NC groups.
4. CC group showed significantly lower *M* than the NC group in both seasons, indicating reduced heat storage in the CC group. Correlation analysis also revealed a moderate correlation between *M* and the cold constitution.
5. Local *t*_*sk*_ values were mostly lower in the CC group compared to the NC group. *t*_*nose*_, *t*_*cheek*_, *t*_*palmar finger*_, and *t*_*dorsal finger*_ were significantly lower in the CC group in the summer. However, in winter, only *t*_*nose*_ and *t*_*cheek*_ significantly differed.
6. *Δt*_*forehead–nose*_ and *Δt*_*palm–palmar finger*_ were significantly higher in the CC group comparing with NC group, which indicates the association of stronger vasoconstriction with cold constitution.

Considering those research findings, it can be stated that cold constitution should be recognized as a key individual aspect when universal thermal comfort is considered.

### Limitations

This research work was performed in an office environment where the environment was thermoneutral. Future studies should investigate a wider variety of thermal environments to gain a more comprehensive understanding of the impact of environmental conditions on the thermal perceptions associated with cold constitution. Additionally, in this research, the number of female participants was lower than that of male participants, which may have affected the present results. Maintaining gender balance, as gender is a significant predictor of cold constitution, could direct a more precise comparison between the CC group and the NC group.

## Data Availability

No datasets were generated or analysed during the current study.
